# Association Between Platelet and Cerebral Small Vessel Disease: A Secondary Analysis Based on a Retrospective Cross‐Sectional Study in Korean Adults

**DOI:** 10.1002/brb3.70771

**Published:** 2025-08-22

**Authors:** Shujuan Huang, Hanbo Chen, Linbo Cai, Zhaoxi Liu, Zhanbo Yu, Yuqin Dan, Danghan Xu, Yunxuan Huang

**Affiliations:** ^1^ Department of Rehabilitation Guang Dong Sanjiu Brain Hospital Guangzhou China; ^2^ Oncology Department The Affiliated Traditional Chinese Medicine Hospital of Guangzhou Medical University Guangzhou China; ^3^ General Medicine Department Ruibao Street Community Health Service Center Guangzhou China; ^4^ School of Rehabilitation Medicine Shandong University of Traditional Chinese Medicine Jinan China; ^5^ Acupuncture Department The First Affiliated Hospital of Guangzhou University of Chinese Medicine Guangzhou China

**Keywords:** cross‐sectional study, cerebral small vessel disease, Korean adults, moderate to severe cerebral white matter hyperintensities, platelet count

## Abstract

**Objective:**

This study aimed to explore an association between platelet count and moderate to severe cerebral white matter hyperintensities (MS‐cWMH), a key imaging marker of cerebral small vessel disease (CSVD), in individuals with cardiovascular risk factors, hypothesizing that higher platelet counts are associated with increased CSVD severity.

**Methods:**

A retrospective cross‐sectional study analyzed data from 1,011 participants aged ≥45 years at CHA Bundang Medical Center in Seoul, Korea, between 2008 and 2014. Participants were selected based on predefined criteria, including cardiovascular risk factors (e.g., hypertension, hyperlipidemia, smoking) and no history of stroke, and underwent brain MRI and MRA as part of the study protocol. Magnetic resonance angiography (MRA) was performed to exclude participants with large vessel disease, such as intracranial or extracranial arterial stenosis. A multivariable logistic regression model was constructed to assess the association between platelet count and MS‐cWMH, incrementally adjusting for demographic characteristics, cardiovascular risk factors, and imaging features.

**Results:**

The study revealed a significant association between platelet count and MS‐cWMH. Each increase of 100×10^9/L in platelet count was associated with a 1.37‐fold increase in the risk of MS‐cWMH (95% CI: 1.06‐1.77, p = 0.0168). The association was more pronounced in females, elderly individuals aged 68–85 years, and patients with diabetes and remained robust in multiple sensitivity analyses.

**Conclusion:**

Higher platelet counts are significantly associated with an increased risk of MS‐cWMH, particularly in specific high‐risk populations such as females, elderly individuals aged 68–85 years, and patients with diabetes. Platelet count may serve as a potential biomarker for assessing the risk of CSVD, aiding in the early identification of high‐risk individuals for preventive interventions. This finding highlights the potential clinical significance of monitoring platelet counts in high‐risk populations. Strategies to reduce high platelet counts, such as antiplatelet therapy (e.g., clopidogrel, aspirin) or lifestyle interventions (e.g., diet and exercise), may help mitigate the risk of MS‐cWMH. Further longitudinal studies are needed to evaluate the effectiveness of these interventions in reducing MS‐cWMH risk. Additionally, future research should investigate the biological mechanisms linking platelet count to CSVD, particularly the roles of platelet activation, inflammation, and endothelial dysfunction, to provide a more comprehensive understanding of this association.

## Graphical abstract

This graphical abstract illustrates the association between platelet count and cerebral small vessel disease (CSVD) severity, specifically measured by magnetic resonance imaging markers of cerebral white matter hyperintensities (MS‐CWMH). Higher platelet counts were associated with increased MS‐CWMH severity, suggesting that platelets may play a crucial role in the development and progression of cerebral small vessel disease. This finding was derived from a secondary analysis of a retrospective cross‐sectional study in Korean adults, providing new insights into the potential mechanisms underlying CSVD pathogenesis.

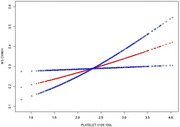

## 1 Introduction

Cerebral Small Vessel Disease (CSVD) is a group of disorders affecting the small arteries, arterioles, and capillaries within the brain. It is highly prevalent among the elderly population and has significant public health implications, largely due to its association with cognitive decline, stroke, and dementia (Wardlaw et al. 2019). Given the aging global population and the increasing prevalence of vascular cognitive impairment, there is an urgent need for biomarkers that can aid in early detection and risk stratification for these conditions (Wardlaw et al. 2019; Debette and Markus 2010). Among these, moderate to severe cerebral white matter hyperintensities (MS‐cWMH) are important radiological markers of CSVD, commonly used to assess the severity and prognosis of the disease (Debette and Markus 2010).

Platelets play a critical role not only in hemostasis but also in vascular endothelial function, inflammatory responses, and thrombogenesis (Pan et al. 2022). While platelet function is crucial in these processes, this study focuses on platelet count due to its simplicity, cost‐effectiveness, and widespread availability in clinical practice (Kaufman et al. 2015). Platelet count serves as a practical biomarker that can be easily integrated into routine risk assessment protocols, particularly in resource‐limited settings (Udeh et al. 2024). These processes are closely linked to the pathophysiology of MS‐cWMH, as inflammation and endothelial dysfunction contribute to blood‐brain barrier disruption, while thrombogenesis promotes microvascular occlusion and ischemia (Jiang et al. 2018). In recent years, an increasing number of studies have indicated that platelets may participate in the pathological processes of CSVD by promoting thrombosis and mediating inflammatory responses. However, the relationship between platelet count and MS‐cWMH remains inconsistent, with some studies finding that elevated platelet counts are associated with an increased risk of CSVD, while others have not observed significant associations (Staszewski et al. 2013; Lavallée et al. 2013). These discrepancies may stem from differences in study design, sample characteristics, and analytical methods.

Therefore, this study aims to investigate the association between platelet count and MS‐cWMH in Korean adults with cardiovascular risk factors. We hypothesize that an increase in platelet count is associated with an increased risk of MS‐cWMH, particularly in specific high‐risk populations. By analyzing cross‐sectional data, this study hopes to provide new biomarkers for the risk assessment of CSVD and offer a theoretical foundation for clinical intervention strategies.

The results of this study may have important clinical significance. If platelet count is indeed associated with the risk of MS‐cWMH, it can serve as a simple and cost‐effective biomarker for the risk assessment and early intervention of CSVD in clinical practice.

## 2 Methods

### 2.1 Data Sources

**FIGURE 1:**
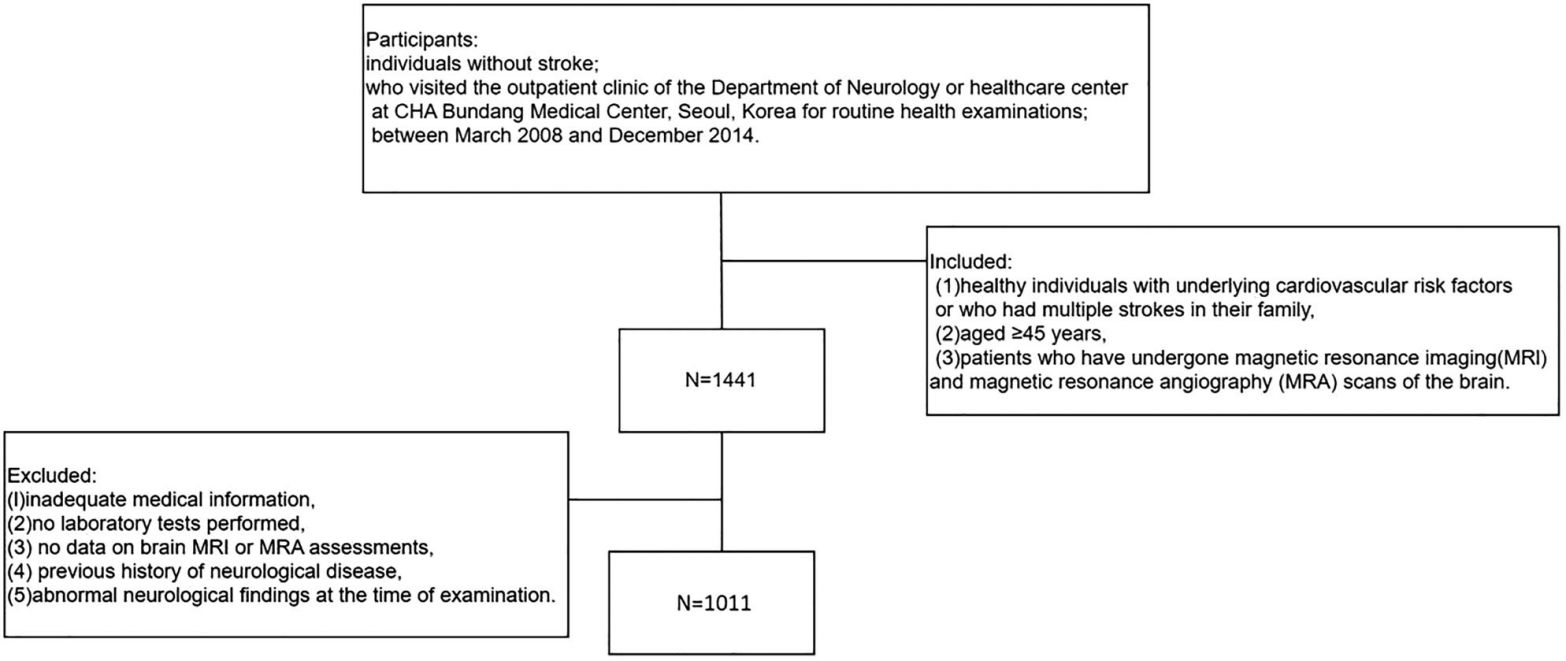
Study procedure.

This secondary analysis utilized data published in a previous study on CSVD. All data were sourced from the comprehensive health database of CHA Bundang Medical Center, which includes clinical and imaging data collected from March 2008 to December 2014. Participants were selected based on predefined criteria to ensure data reliability and relevance (Lee et al. 2015). This study protocol was approved by the Ethics Review Committee of CHA Bundang Medical Center (IRB number BD‐2010‐083), and all procedures comply with the Declaration of Helsinki. Data usage is governed by strict confidentiality agreements to protect participant privacy, and all analyses adhere to relevant data protection regulations. The inclusion criteria were as follows: (1) healthy individuals with underlying cardiovascular risk factors or who had multiple strokes in their family; (2) aged ≥ 45 years, as this age group represents a population at increased risk for CSVD‐related changes based on prior studies (Lee et al. 2015); (3) patients who have undergone magnetic resonance imaging (MRI) and magnetic resonance angiography (MRA) scans of the brain. The exclusion criteria were as follows: (1) inadequate medical information, (2) no laboratory tests performed, (3) no data on brain MRI or MRA assessments, (4) previous history of neurological disease, and (5) abnormal neurological findings at the time of examination (Yao et al. 2022). Out of the initially eligible 1,441 participants, 430 were excluded based on these criteria. A detailed selection process is illustrated in Figure 1. This dataset is representative of the Korean adult population with cardiovascular risk factors, enhancing the external validity of the study results. However, the single‐center design may limit the generalizability of the findings to other populations or healthcare settings.

### 2.2 Variables

The primary exposure variable was platelet count obtained through laboratory tests. The primary outcome variable was moderate‐to‐severe cerebral white matter hyperintensities (MS‐cWMH), which was quantitatively assessed using the validated Fazekas scale on fluid‐attenuated inversion recovery (FLAIR) MRI sequences performed on 1.5T MR systems (Leonards et al. 2012; Fazekas et al. 1987). Brain MRI was performed using 1.5T MR systems, including FLAIR, T1‐weighted, and T2‐weighted sequences (Wardlaw et al. 2013). The severity of cWMH was graded using the Fazekas scale on FLAIR images, with scores of 3–6 indicating moderate to severe changes (Lee et al. 2015; Fazekas et al. 1987). Grading was performed independently by a neurologist and a radiologist, both blinded to clinical data. Potential confounding variables were selected based on previous literature and clinical relevance, including demographic characteristics (age, sex), cardiovascular risk factors (hypertension, diabetes mellitus, hyperlipidemia), laboratory parameters (white blood cell count, hematocrit, platelet count, total cholesterol, triglycerides, and fasting blood glucose), and imaging features (intra‐ and extracranial arterial stenosis).

### 2.3 Statistical Analysis

Continuous variables were expressed as mean ± standard deviation for normally distributed data or median (interquartile range) for skewed distributions, and categorical variables as frequencies (percentages). Between‐group comparisons were performed using Student's t‐test, the Mann‐Whitney U test, or the chi‐square test as appropriate. The association between platelet count and MS‐cWMH was analyzed using multiple approaches. First, we constructed three logistic regression models with progressive adjustment for potential confounders identified from previous literature and clinical relevance: Model 1 (unadjusted); Model 2 (adjusted for age and sex); and Model 3 (additionally adjusted for cardiovascular risk factors, laboratory parameters, and imaging features). To explore potential nonlinear relationships, we employed generalized additive models with smooth curve fitting using penalized splines. When nonlinear relationships were detected, recursive algorithms were used to calculate inflection points and construct piecewise linear regression models; when no nonlinear relationships were detected, linear regression models were directly applied for analysis. For sensitivity analyses, we categorized platelet count into three groups based on predefined clinical criteria and calculated tests for trend. Further details are available in the supplementary materials. Subgroup analyses were performed using stratified regression models, with interaction tests to assess effect modification by pre‐specified variables including age, sex, and cardiovascular risk factors. All statistical analyses were performed using R version 3.4.3 (R Foundation for Statistical Computing, Vienna, Austria). Two‐sided p‐values < 0.05 were considered statistically significant.

## 3 Results

### 3.1 Baseline Characteristics of Participants

Among the 1,011 participants enrolled in this study, platelet counts were categorized into three groups based on predefined clinical criteria: low‐exposure (<1×10^9/100L, n = 3), middle‐exposure (1‐3×10^9/100L, n = 898), and high‐exposure (>3×10^9/100L, n = 110) groups. Significant differences were observed in several baseline characteristics across these groups. The high‐exposure group showed a higher mean age (66.27 ± 8.40 years) compared to the middle‐exposure (63.90 ± 9.18 years) and low‐exposure groups (65.00 ± 10.82 years) (p = 0.036). A notable gender distribution pattern emerged, with female representation increasing from the low‐exposure (0%) to the high‐exposure group (74.5%) (p = 0.005).

Laboratory parameters demonstrated significant intergroup variations. White blood cell count showed a progressive increase across groups (5.41 ± 1.29, 6.44 ± 1.88, and 7.60 ± 1.94 × 10^9/L, respectively; P < 0.001). GOT levels were markedly elevated in the low‐exposure group (57.00 ± 33.87 IU/L) compared to the middle (23.55 ± 9.43 IU/L) and high‐exposure groups (22.90 ± 7.73 IU/L) (P < 0.001). Total cholesterol levels showed an ascending trend from low‐to high‐exposure groups (153.67 ± 89.13, 192.99 ± 38.71, and 201.95 ± 45.79 mg/dL, respectively; p = 0.018). The eGFR was notably higher in the low‐exposure group (99.13 ± 10.25 mL/min/1.73m^2^) compared to other groups (p = 0.022).

Regarding comorbidities, hypertension prevalence increased significantly across groups (33.3%, 55.6%, and 71.8%, respectively; p = 0.004). Notably, the prevalence of MS‐cWMH varied significantly among groups (66.7%, 27.2%, and 43.6%, respectively; P < 0.001), with the low‐exposure group showing the highest prevalence despite its small sample size. The baseline characteristics of the participants are summarized in Table 1.

**TABLE 1:** Baseline characteristics of study population by MS‐cWMH.

	Platelet (×10^9/100L categorical)			
Characteristics	Low‐exposure group(n = 3)	Middle‐exposure group(n = 898)	High‐exposure group(n = 110)	*p*‐value
SBP (mmHg)	146.67 ± 28.87	131.46 ± 18.28	133.24 ± 18.34	0.231
Age (years)	65.00 ± 10.82	63.90 ± 9.18	66.27 ± 8.40	0.036
DBP (mmHg)	83.33 ± 5.77	80.06 ± 11.45	79.77 ± 12.28	0.858
DIFF_BP (mmHg)	63.33 ± 23.09	51.40 ± 14.09	53.46 ± 12.58	0.120
WBC (×10^9/L)	5.41 ± 1.29	6.44 ± 1.88	7.60 ± 1.94	<0.001
Hematocrit (%)	38.37 ± 2.40	40.07 ± 3.99	39.52 ± 5.11	0.335
Fasting glucose (mg/dL)	120.33 ± 50.85	128.38 ± 48.41	125.45 ± 48.21	0.804
Uric acid (mg/dL)	4.67 ± 2.25	4.55 ± 1.42	4.33 ± 1.16	0.292
GOT (IU/L)	57.00 ± 33.87	23.55 ± 9.43	22.90 ± 7.73	<0.001
GPT (IU/L)	39.00 ± 36.86	23.63 ± 18.90	23.56 ± 13.96	0.355
ALP (IU/L)	223.67 ± 33.56	181.44 ± 56.79	195.14 ± 66.09	0.031
Total cholesterol (mg/dL)	153.67 ± 89.13	192.99 ± 38.71	201.95 ± 45.79	0.018
Triglyceride (mg/dL)	121.67 ± 75.05	148.39 ± 96.23	159.31 ± 86.43	0.462
eGFR (mL/min/1.73 m2)	99.13 ± 10.25	74.21 ± 16.83	72.53 ± 16.32	0.022
Sex (%)				0.005
Male	100.0	36.5	25.5	
Female	0.0	63.5	74.5	
Hypertension (%)				0.004
No	66.7	44.4	28.2	
Yes	33.3	55.6	71.8	
Diabetes mellitus (%)				0.174
No	33.3	78.1	77.3	
Yes	66.7	21.9	22.7	
Hyperlipidemia (%)				0.335
No	66.7	67.9	60.9	
Yes	33.3	32.1	39.1	
CAOD (%)				0.687
No	100.0	94.7	96.4	
Yes	0.0	5.3	3.6	
Smoking (%)				0.811
No	66.7	79.6	80.9	
Yes	33.3	20.4	19.1	
Statin medication (%)				0.559
No	100.0	77.7	75.5	
Yes	0.0	22.3	24.5	
SLI (%)				0.151
No	66.7	88.8	83.6	
Yes	33.3	11.2	16.4	
ECAS (%)				0.503
No	66.7	88.4	88.2	
Yes	33.3	11.6	11.8	
ICAS (%)				0.798
No	100.0	89.9	90.9	
Yes	0.0	10.1	9.1	
LCAS (%)				0.821
No	66.7	81.0	80.9	
Yes	33.3	19.0	19.1	
MS‐cWMH (%)				<0.001
No	33.3	72.8	56.4	
Yes	66.7	27.2	43.6	

*Note*: Continuous variables are presented as mean ± standard deviation; categorical variables as percentages. P‐values were derived from one‐way ANOVA for continuous variables (after the normality test) and the chi‐square test or Fisher's exact test (when expected frequencies were <5) for categorical variables. Platelet groups were categorized as: low‐exposure (<1×10^9/100 L), middle‐exposure (1‐3×10^9/100 L), high‐exposure (>3×10^9/100 L).

Abbreviations: ALP, alkaline phosphatase; CAOD, coronary artery occlusive disease; DBP, diastolic blood pressure; DIFF_BP, difference between systolic and diastolic blood pressure; eGFR, estimated glomerular filtration rate; ECAS, extracranial artery stenosis; GOT, glutamic oxaloacetic transaminase; GPT, glutamic pyruvic transaminase; ICAS, intracranial artery stenosis; LCAS, large cerebral artery stenosis; MS‐cWMH, moderate to severe cerebral white matter hyperintensities; SBP, systolic blood pressure; SLI, silent lacunar infarction; WBC, white blood cell count.

### 3.2 Univariate Analysis

Univariate logistic regression analysis identified multiple factors significantly associated with MS‐cWMH (Table 2). Platelet count showed a significant positive association with MS‐cWMH risk (OR = 1.34, 95% CI: 1.06‐1.68, p = 0.0134), with each 100×10^9/L increase in platelet count corresponding to a 34% higher risk. Demographic factors including age (OR = 1.13, 95% CI: 1.11‐1.15, p < 0.0001) and female gender (OR = 1.49, 95% CI: 1.11‐1.99, p = 0.0079) were significantly associated with MS‐cWMH. Among vascular risk factors, hypertension demonstrated the strongest association (OR = 2.07, 95% CI: 1.55‐2.76, p < 0.0001), followed by systolic blood pressure (OR = 1.01, 95% CI: 1.00‐1.02, p = 0.0032) and pulse pressure (OR = 1.02, 95% CI: 1.01‐1.03, p < 0.0001). Diabetes mellitus was also significantly associated with increased MS‐cWMH risk (OR = 1.45, 95% CI: 1.06‐1.99, p = 0.0212). Notably, among cerebrovascular indicators, silent lacunar infarction showed the strongest association (OR = 5.33, 95% CI: 3.57‐7.97, p < 0.0001), while intracranial artery stenosis (OR = 1.95, 95% CI: 1.28‐2.96, p = 0.0019) and large cerebral artery stenosis (OR = 1.60, 95% CI: 1.15‐2.22, p = 0.0054) also demonstrated significant associations. Laboratory parameters including decreased hematocrit (OR = 0.95, 95% CI: 0.92‐0.98, p = 0.0034) and eGFR (OR = 0.97, 95% CI: 0.96‐0.98, p < 0.0001) and increased ALP (OR = 1.00, 95% CI: 1.00‐1.01, p = 0.0025) were significantly associated with MS‐cWMH risk.

**TABLE 2:** Univariate analysis of MS‐cWMH.

	MS‐cWMH	
Variables	OR (95% CI)	*p*‐value
Platelet (×10^9/100L)	1.34 (1.06, 1.68)	0.0134
SBP (mmHg)	1.01 (1.00, 1.02)	0.0032
Sex		
Male	Ref.	
Female	1.49 (1.11, 1.99)	0.0079
Age (years)	1.13 (1.11, 1.15)	<0.0001
DBP (mmHg)	1.00 (0.99, 1.01)	0.7759
DIFF_BP (mmHg)	1.02 (1.01, 1.03)	<0.0001
Hypertension		
No	Ref.	
Yes	2.07 (1.55, 2.76)	<0.0001
Diabetes mellitus		
No	Ref.	
Yes	1.45 (1.06, 1.99)	0.0212
Hyperlipidemia		
No	Ref.	
Yes	1.03 (0.77, 1.38)	0.8302
CAOD		
No	Ref.	
Yes	1.20 (0.66, 2.17)	0.5564
Smoking		
No	Ref.	
Yes	0.77 (0.54, 1.09)	0.1386
Statin medication		
No	Ref.	
Yes	0.92 (0.66, 1.28)	0.6172
SLI		
No	Ref.	
Yes	5.33 (3.57, 7.97)	<0.0001
ECAS		
No	Ref.	
Yes	1.18 (0.78, 1.79)	0.4271
ICAS		
No	Ref.	
Yes	1.95 (1.28, 2.96)	0.0019
LCAS		
No	Ref.	
Yes	1.60 (1.15, 2.22)	0.0054
WBC (×10^9/L)	1.06 (0.98, 1.13)	0.1266
Hematocrit	0.95 (0.92, 0.98)	0.0034
Fasting glucose (mg/dL)	1.00 (1.00, 1.00)	0.2084
Uric acid (mg/dL)	1.02 (0.92, 1.12)	0.7029
GOT (IU/L)	1.01 (1.00, 1.03)	0.0816
GPT (IU/L)	0.99 (0.98, 1.00)	0.2053
ALP (IU/L)	1.00 (1.00, 1.01)	0.0025
Total cholesterol (mg/dL)	1.00 (1.00, 1.00)	0.5395
Triglyceride (mg/dL)	1.00 (1.00, 1.00)	0.1221
eGFR (mL/min/1.73 m2)	0.97 (0.96, 0.98)	<0.0001

*Note*: Univariate logistic regression was used to analyze the association between various factors and MS‐cWMH (moderate to severe cerebral white matter hyperintensities). Results are presented as odds ratios (OR) with 95% confidence intervals (CI). ‘Ref.’ indicates the reference category for categorical variables.

Abbreviations: ALP, alkaline phosphatase; CAOD, coronary artery occlusive disease; DBP, diastolic blood pressure; DIFF_BP, difference between systolic and diastolic blood pressure; ECAS, extracranial artery stenosis; eGFR, estimated glomerular filtration rate; GOT, glutamic oxaloacetic transaminase; GPT, glutamic pyruvic transaminase; ICAS, intracranial artery stenosis; LCAS, large cerebral artery stenosis; SBP, systolic blood pressure; SLI, silent lacunar infarction; WBC, white blood cell count.

### 3.3 Subgroup Analysis Results

Stratified analyses revealed distinct patterns in the association between platelet count and MS‐cWMH across demographic and clinical subgroups (Table 3). The association was particularly pronounced in specific populations: females (OR = 1.34, 95% CI: 1.01‐1.77, p = 0.0436), older adults aged 68–85 years (OR = 1.54, 95% CI: 1.09‐2.18, p = 0.0142), and notably in patients with diabetes (OR = 2.45, 95% CI: 1.46‐4.10, p = 0.0006). The relationship was also significant among participants without hyperlipidemia (OR = 1.48, 95% CI: 1.12‐1.97, p = 0.0061) and those without coronary artery occlusive disease (OR = 1.31, 95% CI: 1.03‐1.65, p = 0.0254). When stratified by metabolic parameters, the association was strongest in the highest tertile of fasting glucose (128‐418 mg/dL; OR = 1.80, 95% CI: 1.16‐2.79, p = 0.0092) and in the lowest tertile of estimated glomerular filtration rate (14.2‐67.1 mL/min/1.73 m2; OR = 1.48, 95% CI: 1.01‐2.18, p = 0.0443). While white blood cell count tertiles showed no significant associations, a trend toward significance was observed in the highest tertile (p = 0.0653). These stratified analyses suggest that the relationship between platelet count and MS‐cWMH is particularly robust in the presence of certain demographic and clinical characteristics, notably advanced age, diabetes, and impaired renal function.

**TABLE 3:** Stratified analysis of the association between platelet count and MS‐cWMH across various demographic and clinical factors.

X = Platelet (×10^9/100L)		MS‐cWMH	
	N	OR (95% CI)	*p*‐value
Sex			
Male	359	1.17 (0.77, 1.78)	0.4663
Female	652	1.34 (1.01, 1.77)	0.0436
Age (years)			
45 ‐ 58	291	0.70 (0.28, 1.76)	0.4451
59 ‐ 67	354	1.43 (0.98, 2.08)	0.0617
68 ‐ 85	366	1.54 (1.09, 2.18)	0.0142
Diabetes mellitus			
No	787	1.13 (0.87, 1.47)	0.3648
Yes	224	2.45 (1.46, 4.10)	0.0006
Hyperlipidemia			
No	679	1.48 (1.12, 1.97)	0.0061
Yes	332	1.05 (0.69, 1.61)	0.8158
CAOD			
No	959	1.31 (1.03, 1.65)	0.0254
Yes	52	2.43 (0.80, 7.38)	0.1162
WBC (×10^9/L)			
1.57 ‐ 5.54	336	1.03 (0.61, 1.76)	0.9028
5.55 ‐ 6.99	338	1.20 (0.76, 1.90)	0.4272
7 ‐ 16.94	337	1.37 (0.98, 1.90)	0.0653
Fasting glucose (mg/dL)			
67 ‐ 98	337	1.10 (0.77, 1.56)	0.6033
99 ‐ 127	334	1.38 (0.90, 2.10)	0.1401
128 ‐ 418	340	1.80 (1.16, 2.79)	0.0092
eGFR (mL/min/1.73 m2)			
14.2 ‐ 67.1	336	1.48 (1.01, 2.18)	0.0443
67.2 ‐ 78.9	338	1.46 (0.98, 2.19)	0.0661
79 ‐ 140	337	0.90 (0.56, 1.46)	0.6778

*Note*: Stratified analysis was performed using logistic regression models to examine the association between platelet count and MS‐cWMH (moderate to severe cerebral white matter hyperintensities) across various subgroups. Results are presented as odds ratios with 95% confidence intervals and *p*‐values. Continuous variables were categorized into tertiles for stratification. *N* represents the number of participants in each subgroup.

Abbreviations: CAOD, coronary artery occlusive disease; eGFR, estimated glomerular filtration rate; WBC, white blood cell count.

### 3.4 The Results of the Relationship Between Platelet and MS‐cWMH

The association between platelet count and MS‐cWMH was examined using multivariate logistic regression with three models: unadjusted and two adjusted models (Table 4). When analyzed as a continuous variable, platelet count maintained a significant positive association with MS‐cWMH across all models, with the fully adjusted model (Adjustment II) showing a 37% increased risk of MS‐cWMH for each 100×10^9/L increase in platelet count (OR = 1.37, 95% CI: 1.06‐1.77, p = 0.0168). In categorical analysis (reference: <1×10^9/100L), although both the ≥1 to ≤3×10^9/100L group (OR = 0.09, 95% CI: 0.01‐1.39, p = 0.0843) and >3×10^9/100L group (OR = 0.15, 95% CI: 0.01‐2.44, p = 0.1809) showed lower point estimates in the fully adjusted model, the wide confidence intervals and non‐significant p‐values preclude definitive conclusions. When analyzed as a categorical continuous variable, the association showed a dose‐dependent pattern, with the strongest relationship in the unadjusted model (OR = 1.93, 95% CI: 1.29‐2.88, p = 0.0013), which remained significant after initial adjustment (OR = 1.67, 95% CI: 1.07‐2.60, p = 0.0232) but attenuated in the fully adjusted model (OR = 1.56, 95% CI: 1.00‐2.45, p = 0.0511).

**TABLE 4:** Multivariable logistic regression analysis of platelet in the presence of MS‐cWMH.

	MS‐cWMH					
	Non‐adjusted		Adjust I		Adjust II	
Logistic regression model	OR (95% CI)	p‐value	OR (95% CI)	p‐value	OR (95% CI)	p‐value
Platelet (×10^9/100 L)	1.34 (1.06, 1.68)	0.0134	1.42 (1.10, 1.83)	0.0073	1.37 (1.06, 1.77)	0.0168
Platelet (×10^9/100 L) categorical						
<1	Ref.		Ref.		Ref.	
≥1, ≤3	0.19 (0.02, 2.07)	0.1712	0.12 (0.01, 1.81)	0.1250	0.09 (0.01, 1.39)	0.0843
>3	0.39 (0.03, 4.40)	0.4439	0.21 (0.01, 3.38)	0.2723	0.15 (0.01, 2.44)	0.1809
Platelet (×10^9/100 L) categorical continuous	1.93 (1.29, 2.88)	0.0013	1.67 (1.07, 2.60)	0.0232	1.56 (1.00, 2.45)	0.0511

*Note*: Multivariable logistic regression analysis was performed to examine the association between platelet count and MS‐cWMH (moderate to severe cerebral white matter hyperintensities). Three models were used: Non‐adjusted, Adjust I, and Adjust II, with increasing levels of adjustment for confounding factors. Results are presented as odds ratios (OR) with 95% confidence intervals (CI) and p‐values. Platelet count was analyzed as a continuous variable, a categorical variable (<1,≥1 to≤3, >3 ×10^9/100 L), and as a categorical continuous variable. ‘Ref.’ indicates the reference category for categorical variables. Specific adjustments for Adjust I and Adjust II models were not provided in the table.

## 4 Discussion

This study investigated the association between platelet count and moderate to severe cerebral white matter hyperintensities (MS‐cWMH) in a large cross‐sectional cohort (n = 1,011). Our findings revealed a significant positive correlation between platelet count and MS‐cWMH risk. After comprehensive adjustment for potential confounders including demographic characteristics, cardiovascular risk factors, and imaging features, each 100×10^9/L increase in platelet count was associated with a 37% higher risk of MS‐cWMH (OR = 1.37, 95% CI: 1.06‐1.77, p = 0.0168) (Greenland 1995). The robustness of this association was demonstrated through three progressive adjustment models: unadjusted, minimally adjusted (age and sex), and fully adjusted (Sterne et al. 2009). Notably, the association remained significant after controlling for established risk factors such as hypertension, diabetes, and hyperlipidemia (Debette and Markus 2010). This finding highlights the potential role of platelet count as an independent risk factor for MS‐cWMH, providing new insights into the pathophysiology of CSVD. Platelet count was chosen as the focus of this study due to its accessibility and clinical relevance. While platelet function plays a critical role in thrombogenesis and inflammation, its assessment requires specialized assays that are not routinely available in clinical practice (Paniccia et al. 2015). Platelet count, on the other hand, is associated with MS‐cWMH and may serve as a simple and cost‐effective indicator for identifying high‐risk individuals. Platelets contribute to MS‐cWMH through multiple mechanisms, including inflammation, endothelial dysfunction, and thrombogenesis (Orian et al. 2021). Inflammatory responses mediated by platelets can exacerbate vascular damage and promote white matter lesions (Rayes et al. 2020). Endothelial dysfunction, a hallmark of CSVD, is closely associated with platelet activation and aggregation, leading to impaired vascular integrity and blood‐brain barrier disruption (Bai et al. 2022)^.^ Thrombogenesis further contributes to microvascular occlusion and ischemia, which are key drivers of MS‐cWMH progression (Markus and de Leeuw 2023). These mechanisms underscore the biological plausibility of our findings and highlight the potential of platelet count as a biomarker for CSVD risk assessment. Given these mechanisms, monitoring platelet counts in high‐risk populations may provide a valuable tool for early identification and risk stratification of CSVD. Furthermore, strategies to reduce elevated platelet counts, such as antiplatelet therapy (e.g., clopidogrel, aspirin) or lifestyle interventions (e.g., diet and exercise), could potentially mitigate the risk of MS‐cWMH (Debette and Markus 2010; Orian et al. 2021). These approaches warrant further longitudinal studies to evaluate their effectiveness in reducing MS‐cWMH risk. Finally, future research should focus on elucidating the biological mechanisms linking platelet count to CSVD, particularly the roles of platelet activation, inflammation, and endothelial dysfunction, to provide a more comprehensive understanding of this association. Additionally, studies exploring platelet function could complement our findings and offer deeper insights into the mechanisms linking platelets to CSVD.

Our findings are consistent with several previous studies, though with some important distinctions. Pan et al. (2022) demonstrated that antiplatelet therapy was associated with improved outcomes in CSVD, and Staszewski et al. (2013) SHEF‐CSVD study supported the relationship between hemostatic factors and CSVD. Our study extends these findings by providing more robust evidence through comprehensive adjustment for potential confounders, including demographic characteristics, cardiovascular risk factors, and imaging features. However, the literature shows some inconsistencies that warrant discussion. Bath et al. (2018) TARDIS trial demonstrated complex relationships between antiplatelet therapy and clinical outcomes in cerebral ischemia, while Vermeer et al. (2003) study emphasized cognitive outcomes in relation to silent brain infarcts. These apparent discrepancies likely reflect methodological differences and varying study populations rather than true contradictions. Notably, while previous studies often focused on community‐based populations, our research specifically targeted individuals with cardiovascular risk factors. The biological plausibility of our findings is supported by several mechanistic studies. Wardlaw et al. (2013) demonstrated that endothelial dysfunction plays a crucial role in CSVD, while Debette et al. (2010) systematic review established the clinical significance of white matter hyperintensities. The work of van Veluw et al. (2017) on cerebral microinfarcts provides a potential mechanistic framework for our observations. Our study makes a distinct contribution by establishing a dose‐dependent relationship between platelet count and MS‐cWMH risk. This finding builds upon previous work by Georgakis et al. (2019) on long‐term outcomes in ischemic stroke and complements Lin et al. (1998) research on observational study methodology while providing more precise risk estimates through comprehensive confounder adjustment.

The clinical value of this study lies in several key aspects. First, our findings establish a dose‐dependent relationship between platelet count and MS‐cWMH risk, providing a potentially valuable biomarker for CSVD risk assessment. This association remained significant after comprehensive adjustment for confounding factors, including demographic characteristics, cardiovascular risk factors, and imaging features (Wardlaw et al. 2013), enhancing the reliability and clinical applicability of our results. Second, our study specifically targeted individuals with cardiovascular risk factors, making the findings particularly relevant for high‐risk populations commonly encountered in clinical practice. This targeted approach, as supported by Wardlaw et al.’s (2019) work on small vessel disease mechanisms, helps bridge the gap between research findings and clinical application. Third, our results provide a theoretical foundation for considering platelet‐related metrics in the clinical management of CSVD. As demonstrated by Debette et al.’s (2010) systematic review, white matter hyperintensities have significant clinical implications, and our findings suggest that platelet count monitoring could be integrated into risk assessment protocols, particularly for elderly patients and those with cardiovascular risk factors. However, several considerations should be noted when translating these findings into clinical practice. As shown in Bath et al. (2018) TARDIS trial, the relationship between platelet‐related interventions and clinical outcomes can be complex. Therefore, while our findings suggest potential therapeutic implications, the clinical application should be cautious and considered alongside other established risk factors. Furthermore, as van Veluw et al. (2017) emphasized, the multifaceted nature of CSVD necessitates a comprehensive approach to risk assessment and management.

This study has several notable strengths. First, we employed a large sample size (n = 1,011) with standardized neuroimaging protocols for small vessel disease research, providing substantial statistical power to detect potential associations between platelet count and MS‐cWMH (Wardlaw et al. 2013). Second, our study comprehensively adjusted for potential confounders through a systematic approach, incorporating demographic characteristics, cardiovascular risk factors, and imaging features, which aligns with current methodological standards for observational studies (Lin et al. 1998).Third, we employed a rigorous statistical analysis strategy. Following established guidelines (Smith et al. 2019), we constructed three progressive models—unadjusted, minimally adjusted, and fully adjusted models—to evaluate the robustness of the association between platelet count and MS‐cWMH. The consistency of results across these models strengthens our confidence in the findings. Fourth, our detailed subgroup analyses revealed important effect modifications by age, sex, and comorbid conditions, particularly in patients with diabetes and impaired renal function (Lee et al. 2015). These findings provide valuable insights for clinical risk stratification and personalized assessment approaches. Finally, we enhanced the reliability of our results through multiple analytical approaches, including sensitivity analyses and different platelet count categorizations (Leonards et al. 2012). This comprehensive analytical strategy, combined with our thorough consideration of potential confounders, strengthens the validity of our findings.

This study has several limitations that warrant discussion. First, due to the cross‐sectional design, we can only establish associations between platelet count and MS‐cWMH rather than determine causality. This limitation is inherent to observational studies, as noted in Wardlaw et al.’s (2013) methodological framework. Second, our study population was restricted to individuals aged ≥45 years with cardiovascular risk factors, potentially limiting the generalizability of our findings. As demonstrated by Staszewski et al. (2013) SHEF‐CSVD study, the relationship between hemostatic factors and CSVD may vary across different demographic groups. Third, while we adjusted for multiple known confounders, the potential influence of unmeasured variables cannot be ruled out, a common challenge in observational studies as highlighted by Lin et al. (1998). These unmeasured factors might include genetic predisposition, environmental exposures, and detailed medication histories. Fourth, our single‐center design and focus on a specific population may affect the external validity of our findings. As Bath et al. (2018) TARDIS trial demonstrated, the complexity of cerebrovascular disease manifestations can vary across different populations and healthcare settings. Finally, we were unable to assess platelet function or other related biomarkers, which, according to van Veluw et al. (2017), could provide additional insights into the mechanisms linking platelets with CSVD. Despite these limitations, our findings provide valuable groundwork for future longitudinal and mechanistic studies.

## Author Contributions

**Shujuan Huang**: writing – original draft, writing – review and editing. **Hanbo Chen**: writing – original draft, writing – review and editing. **Linbo Cai**: data curation, formal analysis. **Zhaoxi Liu**: writing – review and editing, conceptualization. **Zhanbo Yu**: conceptualization, writing – review and editing. **Yuqin Dan**: conceptualization, writing – review and editing. **Danghan Xu**: writing – review and editing, conceptualization, formal analysis. **Yunxuan Huang**: conceptualization, formal analysis, writing – review and editing.

## Ethics Statement

The studies involving human participants were reviewed and approved by the Institutional Review Board (IRB) of CHA Bundang Medical Center (IRB No. BD‐2010‐083). The patients/participants provided their written informed consent to participate in this study.

## Consent

The authors have nothing to report.

## Conflicts of Interest

The authors declare no conflicts of interest.

## Peer Review

The peer review history for this article is available at https://publons.com/publon/10.1002/brb3.70771.

## Supporting information




**Supplementary Materials**: brb370771‐sup‐0001‐SuppMat.pdf

**Supplementary Materials**: brb370771‐sup‐0002‐SuppMat.pdf

## Data Availability

The datasets used and analyzed during the current study are available from the corresponding author on reasonable request.

## References

[brb370771-bib-0001] Bai, T. , S. J. Yu , and J. Feng . 2022. “Advances in the Role of Endothelial Cells in Cerebral Small Vessel Disease.” Frontiers in Neurology 13: 861714. 35481273 10.3389/fneur.2022.861714 PMC9035937

[brb370771-bib-0002] Bath, P. M. , L. J. Woodhouse , J. P. Appleton , et al. 2018. “Antiplatelet Therapy With Aspirin, Clopidogrel, and Dipyridamole versus Clopidogrel Alone or Aspirin and Dipyridamole in Patients With Acute Cerebral Ischaemia(TARDIS): A Randomised,Open‐Label,Phase 3 Superiority Trial.” Lancet 391, no. 10123: 850–859. 29274727 10.1016/S0140-6736(17)32849-0 PMC5854459

[brb370771-bib-0003] Debette, S. , and H. S. Markus . 2010. “The Clinical Importance of White Matter Hyperintensities on Brain Magnetic Resonance Imaging: Systematic Review and Meta‐Analysis.” BMJ 341: c3666.–c. 20660506 10.1136/bmj.c3666 PMC2910261

[brb370771-bib-0004] Fazekas, F. , J. Chawluk , A. Alavi , H. Hurtig , and R. Zimmerman . 1987. “MR Signal Abnormalities at 1.5 T in Alzheimer's Dementia and Normal Aging.” AJR American Journal of Roentgenology 149, no. 2: 351–356. 3496763 10.2214/ajr.149.2.351

[brb370771-bib-0005] Georgakis, M. K. , M. Duering , J. M. Wardlaw , and M. Dichgans . 2019. “WMH and Long‐Term Outcomes in Ischemic Stroke: A Systematic Review and Meta‐Analysis.” Neurology 92, no. 12: e1298–e1308. 30770431 10.1212/WNL.0000000000007142

[brb370771-bib-0006] Greenland, S. 1995. “Dose‐Response and Trend Analysis in Epidemiology: Alternatives to Categorical Analysis.” Epidemiology (Cambridge, Mass.) 6, no. 4: 356–365. 7548341 10.1097/00001648-199507000-00005

[brb370771-bib-0007] Jiang, X. , A. V. Andjelkovic , L. Zhu , et al. 2018. “Blood‐Brain Barrier Dysfunction and Recovery After Ischemic Stroke.” Progress in Neurobiology 163‐164: 144–171. 10.1016/j.pneurobio.2017.10.001 PMC5886838 28987927

[brb370771-bib-0008] Kaufman, R. M. , B. Djulbegovic , T. Gernsheimer , et al. 2015. “Platelet Transfusion: A Clinical Practice Guideline from the AABB.” Annals of Internal Medicine 162, no. 3: 205–213. 25383671 10.7326/M14-1589

[brb370771-bib-0009] Lavallée, P. C. , J. Labreuche , D. Faille , et al. 2013. “Circulating Markers of Endothelial Dysfunction and Platelet Activation in Patients With Severe Symptomatic Cerebral Small Vessel Disease.” Cerebrovascular Diseases (Basel, Switzerland) 36, no. 2: 131–138. 24029712 10.1159/000353671

[brb370771-bib-0010] Lee, H. B. , J. Kim , S. H. Kim , S. Kim , O. J. Kim , and S. H. Oh . 2015. “Association Between Serum Alkaline Phosphatase Level and Cerebral Small Vessel Disease.” PLoS ONE 10, no. 11: 1–11. 10.1371/journal.pone.0143355 PMC4651565 26580067

[brb370771-bib-0011] Leonards, C. O. , N. Ipsen , U. Malzahn , J. B. Fiebach , M. Endres , and M. Ebinger . 2012. “White Matter Lesion Severity in Mild Acute Ischemic Stroke Patients and Functional Outcome After 1 Year.” Stroke 43, no. 11: 3046–3051. 22935398 10.1161/STROKEAHA.111.646554

[brb370771-bib-0012] Lin, D. Y. , B. M. Psaty , and R. A Kronmal . 1998. “Assessing the Sensitivity of Regression Results to Unmeasured Confounders in Observational Studies.” Biometrics 54, no. 3: 948. 9750244

[brb370771-bib-0013] Markus, H. S. , and F. E. de Leeuw . 2023. “Cerebral Small Vessel Disease: Recent Advances and Future Directions.” International Journal of Stroke 18, no. 1: 4–14. 36575578 10.1177/17474930221144911 PMC9806465

[brb370771-bib-0014] Orian, J. M. , C. S. D'Souza , P. Kocovski , et al. 2021. “Platelets in Multiple Sclerosis: Early and Central Mediators of Inflammation and Neurodegeneration and Attractive Targets for Molecular Imaging and Site‐Directed Therapy.” Frontiers in Immunology 12: 620963. 33679764 10.3389/fimmu.2021.620963 PMC7933211

[brb370771-bib-0015] Pan, D. , X. Rong , H. Li , et al. 2022. “Anti‐Platelet Therapy Is Associated With Lower Risk of Dementia in Patients With Cerebral Small Vessel Disease.” Frontiers in Aging Neuroscience 14: 788407. 35431899 10.3389/fnagi.2022.788407 PMC9008232

[brb370771-bib-0016] Paniccia, R. , R. Priora , A. Alessandrello Liotta , and R. Abbate . 2015. “Platelet Function Tests: A Comparative Review.” Vascular Health and Risk Management 11: 133. 25733843 10.2147/VHRM.S44469 PMC4340464

[brb370771-bib-0017] Rayes, J. , J. H. Bourne , A. Brill , and S. P. Watson . 2020. “The Dual Role of Platelet‐Innate Immune Cell Interactions in Thrombo‐Inflammation.” Research and Practice in Thrombosis and Haemostasis 4, no. 1: 23–35. 31989082 10.1002/rth2.12266 PMC6971330

[brb370771-bib-0018] Smith, E. E. , G. J. Biessels , F. De Guio , et al. 2019. “Harmonizing Brain Magnetic Resonance Imaging Methods for Vascular Contributions to Neurodegeneration.” Alzheimer's & Dementia (Amsterdam, Netherlands) 11: 191–204. 10.1016/j.dadm.2019.01.002 PMC6396326 30859119

[brb370771-bib-0019] Staszewski, J. , R. Piusińska‐Macoch , E. Skrobowska , et al. 2013. “Significance of Haemodynamic and Haemostatic Factors in the Course of Different Manifestations of Cerebral Small Vessel Disease: The SHEF‐CSVD Study—Study Rationale and Protocol.” Neuroscience Journal 2013: 1–9. 10.1155/2013/424695 PMC4437267 26317092

[brb370771-bib-0020] Sterne, J. A. C. , I. R. White , J. B. Carlin , et al. 2009. “Multiple Imputation for Missing Data in Epidemiological and Clinical Research: Potential and Pitfalls.” BMJ 338, no. jun29 1: b2393.–b. 19564179 10.1136/bmj.b2393 PMC2714692

[brb370771-bib-0021] Udeh, P. I. , A. M. Olumodeji , T. O. Kuye‐Kuku , O. O. Orekoya , O. Ayanbode , and A. O. Fabamwo . 2024. “Evaluating Mean Platelet Volume and Platelet Distribution Width as Predictors of Early‐Onset Pre‐Eclampsia: A Prospective Cohort Study. Maternal Health.” Neonatology and Perinatology 10, no. 5: 1–8. 10.1186/s40748-024-00174-8 PMC10905831 38424566

[brb370771-bib-0022] van Veluw, S. J. , A. Y. Shih , E. E. Smith , et al. 2017. “Detection, Risk Factors, and Functional Consequences of Cerebral Microinfarcts.” The Lancet Neurology 16, no. 9: 730–740. 28716371 10.1016/S1474-4422(17)30196-5 PMC5861500

[brb370771-bib-0023] Vermeer, S. E. , N. D. Prins , T. Den Heijer , A. Hofman , P. J. Koudstaal , and M. M. B. Breteler . 2003. “Silent Brain Infarcts and the Risk of Dementia and Cognitive Decline.” New England Journal of Medicine 348, no. 13: 1215–1222. 12660385 10.1056/NEJMoa022066

[brb370771-bib-0024] Wardlaw, J. M. , C. Smith , and M. Dichgans . 2019. “Small Vessel Disease: Mechanisms and Clinical Implications.” The Lancet Neurology 18, no. 7: 684–696. 31097385 10.1016/S1474-4422(19)30079-1

[brb370771-bib-0025] Wardlaw, J. M. , E. E. Smith , G. J. Biessels , et al. 2013. “Neuroimaging Standards for Research Into Small Vessel Disease and Its Contribution to Ageing and Neurodegeneration.” The Lancet Neurology 12, no. 8: 822–838. 23867200 10.1016/S1474-4422(13)70124-8 PMC3714437

[brb370771-bib-0026] Yao, T. , A. Di , J. Li , et al. 2022. “Association Between Serum Uric Acid and Intracranial Arterial Stenosis in a Korean Population: A Secondary Analysis Based on a Cross‐Sectional Study.” Frontiers in Neurology 13: 791456. 35359641 10.3389/fneur.2022.791456 PMC8962189

