# Supplementary material for: Association Between Platelet and Cerebral Small Vessel Disease: A Secondary Analysis Based on a Retrospective Cross‐Sectional Study in Korean Adults
Source: Brain Behav. 2025 Aug 22;15(8):e70771. doi: 10.1002/brb3.70771 (PMC12373706; doi:10.1002/brb3.70771)
Supplement: Supplementary file 1 — Supplementary Materials: brb370771‐sup‐0001‐SuppMat.pdf [file BRB3-15-e70771-s001.pdf]

Supplement 1: Association between platelet count and microstructural changes-cerebral white matter hyperintensities (MS-CWMH)

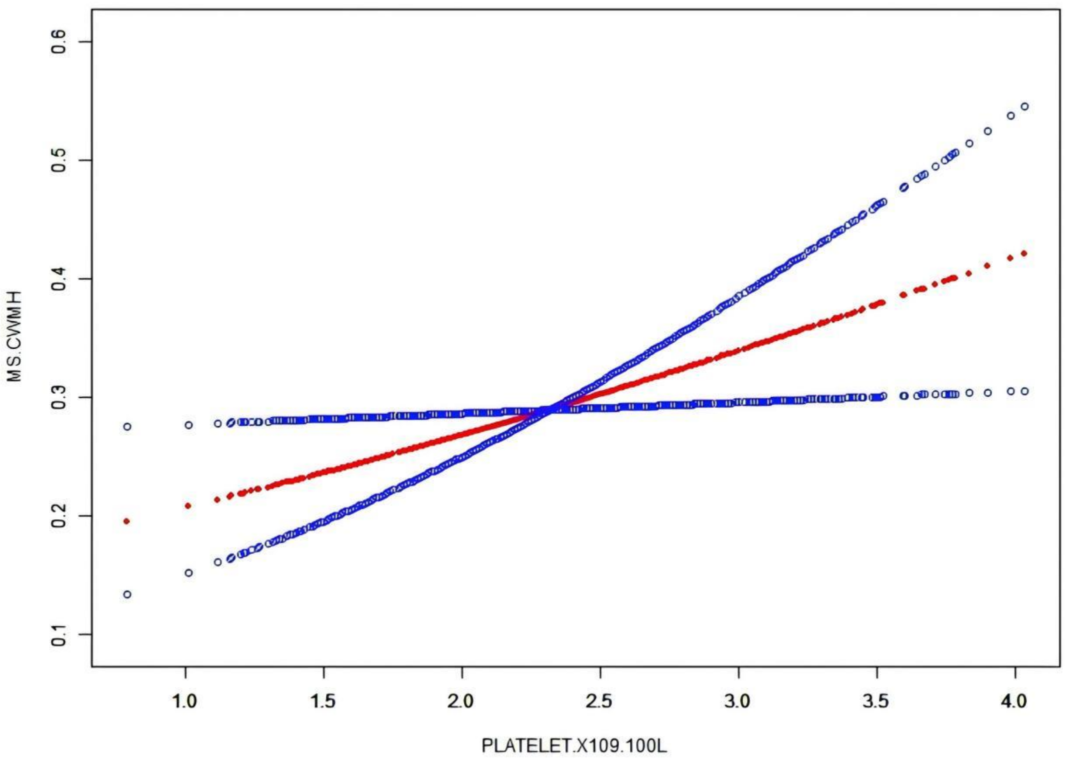

The figure illustrates the relationship between platelet count (x10^9/100L) and the severity of microstructural changes in white matter hyperintensities (MS-CWMH). Blue circles represent individual data points. The red line indicates the smoothed trend of the relationship, while the blue lines depict the 95% confidence interval. A positive linear association is observed across the entire range of platelet counts, with MS-CWMH values increasing as platelet counts rise. The widening of the confidence interval at higher platelet counts suggests increased variability in MS-CWMH at these levels. This relationship implies that elevated platelet counts may be associated with more severe white matter changes, potentially indicating a role for platelets in the progression of cerebral small vessel disease.

Supplement 2: Sensitivity analysis of platelet count categories : comparison of unadjusted and adjusted Models

Exposure	Non-adjusted	Adjust I	Adjust II
PLATELET.X109.100L categorical			
<1	1.0	1.0	1.0
≥1, ≤3	0.19 (0.02, 2.07) 0.1712	0.12 (0.01, 1.81) 0.1250	0.09 (0.01, 1.39) 0.0843
>3	0.39 (0.03, 4.40) 0.4439	0.21 (0.01, 3.38) 0.2723	0.15 (0.01, 2.44) 0.1809

This table presents a sensitivity analysis of the association between platelet count categories and the outcome of interest. Platelet counts (x10^9/100L) were categorized into three groups: <1 (reference), ≥1 to≤3, and >3. Results are shown as odds ratios with 95% confidence intervals and p-values. Three logistic regression models were employed: Non-adjusted (unadjusted), Adjust I (adjusted for primary confounders), and Adjust II (adjusted for additional covariates). The analysis demonstrates the consistency of the association across different adjustment levels, with all models suggesting a potential protective effect of higher platelet counts, although not reaching statistical significance (all p>0.05).
