# Supplementary material for: Association Between Platelet and Cerebral Small Vessel Disease: A Secondary Analysis Based on a Retrospective Cross‐Sectional Study in Korean Adults
Source: Brain Behav. 2025 Aug 22;15(8):e70771. doi: 10.1002/brb3.70771 (PMC12373706; doi:10.1002/brb3.70771)
Supplement: Supplementary file 2 — Supplementary Materials: brb370771‐sup‐0002‐SuppMat.pdf [file BRB3-15-e70771-s002.pdf]

Supplement 3

Table 1| Baseline characteristics of study population by MS-cWMH

Characteristics	Platelet (×10^9/100L)			P-value
	Low-exposure group(n=334)	Middle-exposure group(n=332)	High-exposure group(n=345)	
SBP (mmHg)	131.88 ± 18.21	131.16 ± 17.65	132.04 ± 19.09	0.803
Age (years)	65.09 ± 9.54	63.77 ± 8.71	63.65 ± 9.06	0.076
DBP (mmHg)	80.27 ± 11.24	79.54 ± 11.21	80.30 ± 12.08	0.628
DIFF_BP (mmHg)	51.61 ± 13.49	51.62 ± 14.04	51.74 ± 14.40	0.991
WBC (×10^9/L)	6.11 ± 1.81	6.51 ± 1.83	7.06 ± 1.98	<0.001
Hematocrit (%)	40.20 ± 4.23	40.05 ± 3.64	39.77 ± 4.46	0.396
Fasting glucose (mg/dL)	130.52 ± 49.44	129.16 ± 49.73	124.55 ± 45.83	0.240
Uric acid (mg/dL)	4.57 ± 1.50	4.45 ± 1.40	4.55 ± 1.28	0.522
GOT (IU/L)	24.83 ± 11.38	22.90 ± 7.30	23.02 ± 9.40	0.013
GPT (IU/L)	25.27 ± 26.54	22.66 ± 13.03	23.08 ± 12.40	0.146
ALP (IU/L)	175.39 ± 53.36	184.11 ± 53.39	189.46 ± 65.35	0.006
Total cholesterol (mg/dL)	186.60 ± 37.09	196.39 ± 38.47	198.41 ± 42.67	<0.001
Triglyceride (mg/dL)	139.86 ± 91.40	147.77 ± 95.46	160.50 ± 97.55	0.017
eGFR (mL/min/1.73 m2)	74.59 ± 17.37	73.63 ± 15.20	74.09 ± 17.75	0.759
Platelet (×10^9/100L)	1.75 ± 0.26	2.28 ± 0.12	2.94 ± 0.49	<0.001
Sex (%)				<0.001
Male	44.6	35.5	26.7	
Female	55.4	64.5	73.3	
Hypertension (%)				0.051
No	46.7	44.0	37.7	
Yes	53.3	56.0	62.3	
Diabetes mellitus (%)				0.874
No	78.7	77.7	77.1	
Yes	21.3	22.3	22.9	
Hyperlipidaemia (%)				0.002
No	72.8	68.7	60.3	
Yes	27.2	31.3	39.7	
CAOD (%)				0.060
No	92.5	96.1	95.9	
Yes	7.5	3.9	4.1	
Smoking (%)				0.133
No	76.3	82.5	80.3	
Yes	23.7	17.5	19.7	
Statin medication (%)				0.359

No	79.9	77.4	75.4	
Yes	20.1	22.6	24.6	
SLI (%)				0.723
No	89.2	88.0	87.2	
Yes	10.8	12.0	12.8	
ECAS (%)				0.617
No	88.9	89.2	87.0	
Yes	11.1	10.8	13.0	
ICAS (%)				0.932
No	89.5	90.4	90.1	
Yes	10.5	9.6	9.9	
LCAS (%)				0.868
No	81.4	81.3	80.0	
Yes	18.6	18.7	20.0	
MS-cWMH (%)				0.594
No	72.5	71.4	69.0	
Yes	27.5	28.6	31.0	

Continuous variables are presented as mean ± standard deviation; categorical variables as percentages. P-values were derived from ANOVA for continuous variables and chi-square test for categorical variables. Platelet count groups: Low-exposure (<2.08 ×10^9/100L), Middle-exposure (2.08-2.49 ×10^9/100L), High-exposure (>2.49 ×10^9/100L).

Abbreviations: MS-cWMH (Moderate to Severe Cerebral White Matter Hyperintensities), SBP (Systolic Blood Pressure), DBP (Diastolic Blood Pressure), DIFF_BP (Difference between Systolic and Diastolic Blood Pressure), WBC (White Blood Cell Count), GOT (Glutamic Oxaloacetic Transaminase), GPT (Glutamic Pyruvic Transaminase), ALP (Alkaline Phosphatase), eGFR (estimated Glomerular Filtration Rate), CAOD (Coronary Artery Occlusive Disease), SLI (Silent Lacunar Infarction), ECAS (Extracranial Artery Stenosis), ICAS (Intracranial Artery Stenosis), LCAS (Large Cerebral Artery Stenosis).
